# Simplification of Arboreal Marsupial Assemblages in Response to Increasing Urbanization

**DOI:** 10.1371/journal.pone.0091049

**Published:** 2014-03-07

**Authors:** Bronwyn Isaac, John White, Daniel Ierodiaconou, Raylene Cooke

**Affiliations:** 1 School of Life and Environmental Sciences, Deakin University, Burwood, Victoria, Australia; 2 School of Life and Environmental Sciences, Deakin University, Warrnambool, Victoria, Australia; Institut Pluridisciplinaire Hubert Curien, France

## Abstract

Arboreal marsupials play an essential role in ecosystem function including regulating insect and plant populations, facilitating pollen and seed dispersal and acting as a prey source for higher-order carnivores in Australian environments. Primarily, research has focused on their biology, ecology and response to disturbance in forested and urban environments. We used presence-only species distribution modelling to understand the relationship between occurrences of arboreal marsupials and eco-geographical variables, and to infer habitat suitability across an urban gradient. We used post-proportional analysis to determine whether increasing urbanization affected potential habitat for arboreal marsupials. The key eco-geographical variables that influenced disturbance intolerant species and those with moderate tolerance to disturbance were natural features such as tree cover and proximity to rivers and to riparian vegetation, whereas variables for disturbance tolerant species were anthropogenic-based (e.g., road density) but also included some natural characteristics such as proximity to riparian vegetation, elevation and tree cover. Arboreal marsupial diversity was subject to substantial change along the gradient, with potential habitat for disturbance-tolerant marsupials distributed across the complete gradient and potential habitat for less tolerant species being restricted to the natural portion of the gradient. This resulted in highly-urbanized environments being inhabited by a few generalist arboreal marsupial species. Increasing urbanization therefore leads to functional simplification of arboreal marsupial assemblages, thus impacting on the ecosystem services they provide.

## Introduction

Anthropogenic impacts on biodiversity have been widespread and diverse. Of the many types of anthropogenic disturbance, urbanization, due to its intensity and degree of change to the landscape is considered the most detrimental to biodiversity [1]. Worldwide, urban environments are similar in structure and function as they are continuously modified to meet a narrow set of human requirements [2].

Urban environments generally contain patches of remnant vegetation, highly isolated from each other by a modified matrix, making the remnants susceptible to significant edge effects [3], [4]. These patches rarely contain the full complement of floral and faunal communities present in natural environments, and are susceptible to invasion by non-native species [5]–[7]. As urbanization intensifies there is often a decrease in the abundance of species with specialist habitat and dietary requirements, concurrently those with flexible requirements often increase in abundance and occasionally dominate urban ecosystems [8].

The transition from diverse, native dominated, species assemblages into those dominated by a few highly abundant species has been referred to broadly as biotic homogenization [2]. Since its inception, biotic homogenization has been refined to incorporate the concept that homogenization occurs at multiple scales, including genetic, taxonomic and functional [9]. Taxonomic homogenization infers that species assemblages become more similar over time and space, in response to landscape change or the influences of invasive species [9]. Functional homogenization, alternatively, refers to the roles of species assemblages becoming similar due to the loss of species with unique functional roles [9], [10].

Arboreal marsupials are functionally important in Australian environments, providing ecosystem services such as pollination, fertilization of soils and transportation of seeds [11]. Arboreal marsupials also contribute to trophic structuring by regulating populations of invertebrates and plants, and serving as prey for higher-order carnivores [11], [12]. Australia’s arboreal marsupials range in size from the feathertail glider (*Acrobates pigmaeus*) at 10 g up to Bennett’s tree kangaroo (*Dendrolagus bennettianus*) at 13.5 kg [13]. Possums and gliders are a subset of arboreal marsupials that range in size from the feathertail glider at 10 g through to the mountain brushtail possum (*Trichosurus cunninghami*) at 4.5 kg [13].

Thirteen species of arboreal marsupials inhabit Victoria, Australia, with six as the focus for this research. The greater glider (*Petauroides volans*), mountain brushtail possum and yellow-bellied glider (*Petaurus australis*) are largely confined to wetter, taller continuous tracts of forests [14], [15]. These species are highly susceptible to anthropogenic impacts because they require tree cavities for breeding, exist at low densities, have large spatial requirements, relatively low fecundity and specialist foraging requirements [16]–[19].

The sugar glider (*Petaurus breviceps*) is a dietary specialist that relies on tree exudates and has larger spatial requirements than the mountain brushtail possum. It is, however, less susceptible to anthropogenic impacts and can be observed in forest, agricultural roadsides and some suburban areas [20]. Within these environments, this species has been associated with mixed multi-age forests with intact canopy cover [17], [21]. Sugar gliders require tree cavities for nesting and denning, but in the absence of tree cavities they will use dreys (a spherical stick and leaf lined nest) constructed by the common ringtail possum (*Pseudocheirus peregrinus*), nest boxes and other alternative structures [17], [22].

The common ringtail possum and common brushtail possum (*Trichosurus vulpecula*) have the largest distribution of arboreal marsupials in Australia, inhabiting forests, woodlands, urban parks and streetscapes [23], [24]. This distribution may be associated with the flexibility exhibited in their den site use and diet [20], [25]–[27].

To date, research on the response of possums and gliders to anthropogenic disturbances has focused on human impacts in forests (e.g. altered fire frequency and logging), as well as their persistence in patchy agricultural and urban environments [21], [25], [27]–[29]. There has been limited investigation into the response of possums and gliders to increasing levels of urbanization across a gradient. Urbanization gradients encompass an urbanized core (e.g. cities and city parks), transition through the urban-fringe and into natural forests. Prior research on arboreal marsupials suggests that tolerance to anthropogenic disturbance is linked to patch metrics (e.g. patch size, shape, connectivity and edge effects) [16], [19], habitat structure [16], [17], food [18], [26] and availability of den sites [29], [30].

Based on *a priori* knowledge of arboreal marsupial habitat within forested regions, landscape composition, in particular tree cover and canopy cover, is likely to be a key attribute driving the occurrence of arboreal marsupials. We would expect that complex environments (e.g. forests), with structurally diverse vegetation, well-established canopies and native flowering understories, will maintain diverse arboreal marsupial assemblages. As the complexity of forested systems declines there should be a concurrent change in the diversity of arboreal marsupial assemblages with a reduction in potential habitat. Adaptable arboreal marsupials may initially exhibit increases in moderately urbanized environments, due to the availability of supplementary food and den sites. Further increases in the intensity of urbanization are likely to dramatically alter habitat composition to the point that areas are no longer able to support the ecological requirements of even the most adaptable arboreal marsupials. Each species in an assemblage will have a threshold to disturbance and once this threshold is exceeded the species will become extinct in that area [31]. Urbanization is a landscape changing process and would therefore be expected to lead to restructuring of arboreal marsupial communities. In this research we aim to identify how potential habitat for arboreal marsupial assemblages changes across an urban to forest gradient. We hypothesize that sensitivity to disturbance will be a significant driver of the distribution of species across the urban gradient. We predict that highly sensitive species will be lost from environments at low levels of disturbance and adaptable species will respond positively to initial increases in urbanization, but will be negatively affected by extreme urbanization.

## Methods

### Ethics Statement

This research and all data collected/used were approved under the Victorian Government’s, Department of Environment and Primary Industries (DEPI) in addition to Deakin University’s Animal Ethics Committee (AEC). This research was conducted under DEPI permit number 10003890, with ethics approval A55/2006 from Deakin University.

### Study Site

**Figure 1:**
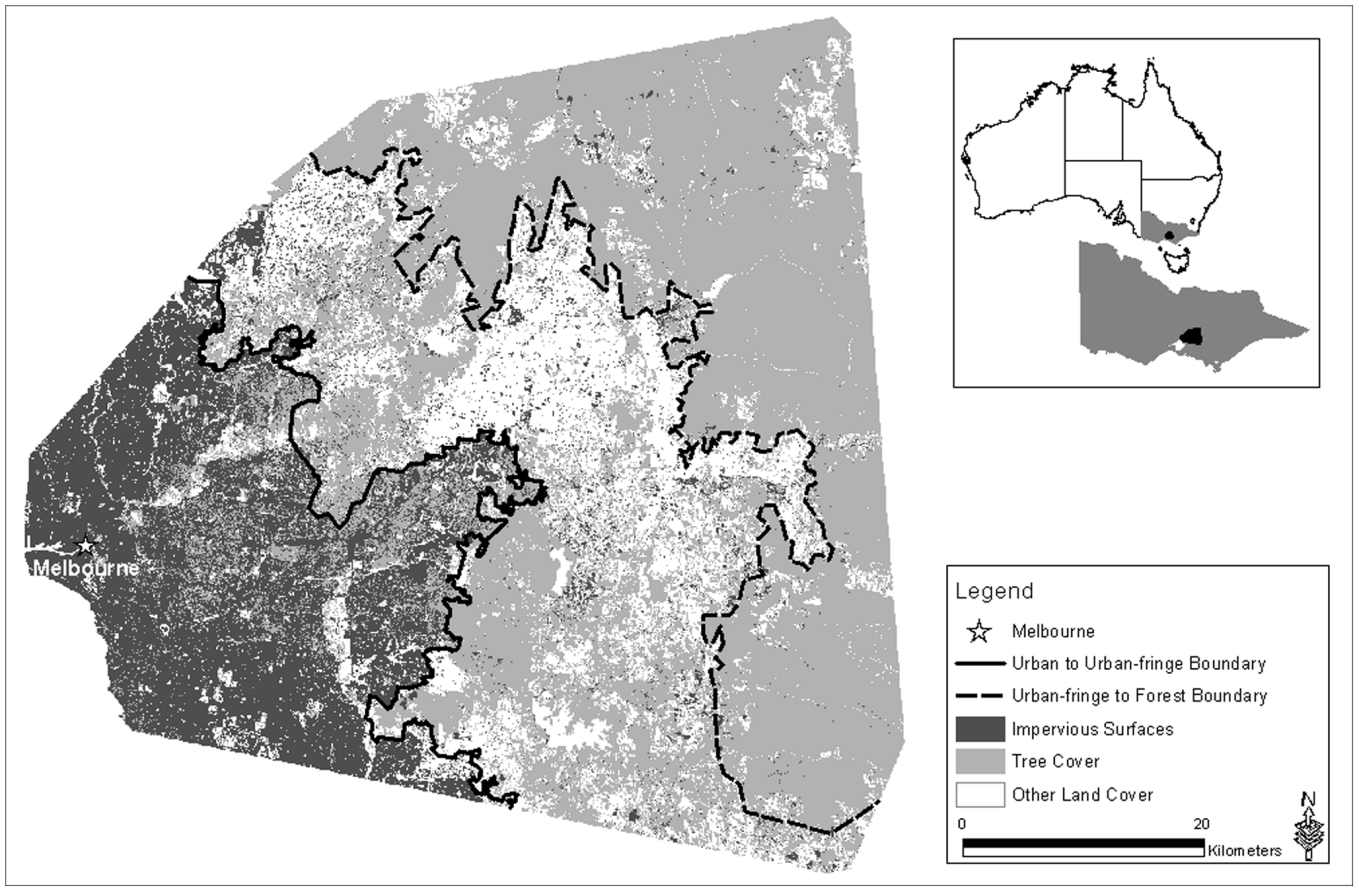
Urban to forest gradient in south eastern Australia used for modelling habitat suitability for arboreal marsupials. The other land cover is predominantly agriculture and other grassed environments, with interspersed rivers and water bodies.

This study was located in south-eastern Victoria, Australia and covered approximately 372,136 ha. The area represents an urban gradient, starting with an urbanized core, that transitions to the urban-fringe through to naturally forested environments (Figure 1). We established boundaries between the urban to urban-fringe and urban-fringe to forest. Boundaries were used to establish clear transitional zones for post-proportional analysis. We digitized these boundaries based on coverage of impervious surfaces and trees using a land cover layer we derived from SPOT 5 satellite imagery (Systèm Pour l’Observation de la Terre) (Figure 1) (Supplementary S1). We classified 20 m×20 m pixels as impervious surfaces, tree cover, grass/agriculture, water or rivers based on spectral reflectance values. We focused on a 20 m×20 m pixel size because lower levels of resolution failed to adequately capture variation in land-use types across the complete gradient.

The study area we selected represents a forest/woodland ecoregion that includes the Gippsland Plains and Highlands-southern fall bioregions and covers a spectrum of environments from urbanised through to relatively intact forest. The area to the west of Melbourne was not surveyed as it falls in the Victorian Volcanic Plains bioregion and is naturally dominated by grassland ecosystems and as such does not provide a useful comparison for investigating arboreal marsupials. The predominant land cover within the urban zone was impervious surface, interspersed with remnant tree cover and other land uses (Figure 1). As the distance from Melbourne increased, land cover composition changed, to produce an urban-fringe or transitional zone. These urban-fringe environments were characterized by multiple land uses such as urban developments, market gardens, remnant vegetation and areas of intense agriculture. Forest environments are located furthest from Melbourne, with land use centred on native forests, sparsely interspersed with agriculture and impervious surfaces (Figure 1).

### Study Species

From the 13 species of possums and gliders known to inhabit Victoria we selected a subset of six for our analysis (Table 1, [13], [14], [27], [30], 32–39]). The distributions of the squirrel glider (*Petaurus norfolcensis*), western pygmy possum (*Cercartetus concinnus*), mountain pygmy possum (*Burramys parvus*) and little pygmy possum (*Cercartetus Lepidus*) were outside of our study area [13]. The eastern pygmy possum (*Cercartetus nanus*) and the feathertail glider (*Acrobates pygmaeus*) were also not used in our research because these species are difficult to detect using spotlighting [14], [32]. We also excluded leadbeaters possum (*Gymnobelideus leadbeateri*) as a suitable candidate for presence-only modelling because a single isolated population of this species occurs within the broad study area and snag watching is a more effective census technique for this species [14], [32]. We therefore collected occurrence data for six species, these being the greater glider, yellow-bellied glider, mountain brushtail possum, sugar glider, common ringtail possum and common brushtail possum.

**Table 1:** Possum and glider species of Victoria with pertinent ecological information.

Species	Diet	Habitat	Home-range	Conservation issues	Functional role
Mountain brushtail possum(*Trichosurus cunninghami*)	Silver wattle *(Acacia* *dealbata*) &fungi	Tall, wet *Eucalyptus* forests	6 ha	Tree hollows, prefers trees withmultiple hollows, habitat loss &modification	Folivore
Common brushtail possum(*Trichosurus vulpecular*)	Leaves, roots, fruit,flowers, grasses &fungi	Forest, woodlands, urbanparks & gardens.	0.35 to 3.08 ha^1^2.03 to 8.61 ha^2^	Introduced predators	Generalistfolivore/omnivore
Common ringtail possum(*Pseudocheirus peregrinus*)	Broad range of plantspecies	Forest, woodlands, coastal teatree, urban parks & gardens.	1 to 2 ha	Introduced predators	Specialist folivore
Little pygmy possum(*Cercartetus Lepidus*)	Nectar, pollen, smallvertebrates &invertebrates	Mallee environments inNorthern Victoria	Drifting home-range	Low abundance, cryptic nature &restricted range	Exudivore
Western Pygmy Possum(*Cercartetus concinnus*)	Nectar and arthropods	Woodlands & heathlands innorthwest Victoria	Unknown	Contraction of range	Exudivore
Eastern pygmy possum(*Cercartetus nanus*)	Nectar, pollen,invertebrates & smallvertebrates	Wet forests, Woodlands,coastal & montaneheathlands	0.24 to 1.7 ha^3^0.18 to 0.61 ha^4^	Small, secretive susceptible to habitat loss & degradation	Exudivore
Mountain pygmy possum(*Burramys parvus*)	Seeds, invertebrates,small vertebrates,arthropods & theBogong moth(*Agrotis infusa*)	Alpine & sub-alpineheathlands above 1,430 m.Rock screes &boulder-fields	0.72 to 5.27 ha	Restricted distribution, introducedherbivores, predators and weeds.Human ski resorts & global warming	Insectivore
Feathertail glider(*Acrobates pygmaeus*)	Nectar, manna, sap,blossoms and insects	*Eucalyptus* forests andwoodlands	0.4 to 2.1 ha	Loss and modification of habitat	Exudivore
Leadbeater’s possum(*Gymnobelideus leadbeateri*)	Exudates, invertebratesand nectar.	Tall wet forests, lowlandswamp woodland and sub-alpine woodland	1 to 3 ha	Specific habitat requirements includelarge diameter trees with hollows,dense *Acacia* understory for food &movement. Susceptible to habitat loss& modification	Exudivore
Sugar glider*(Petaurus breviceps*)	Nectar, pollen, sap,*Acacia* gum, honeydewmanna & invertebrates	Dry sclerophyll forest,Coastal Eucalypt/Banksiaforest & woodland	6.2 to 6.7 ha	Tree hollows, specialized diet &introduced predators	Exudivore
Squirrel glider(*Petaurus norfolcensis*)	Sap & nectar	Dry forest & woodlands	3.1 to 8.8 ha	Small isolated patchy distribution,clearing, natural senescence withoutregeneration & tree hollows	Exudivore
Yellow-bellied glider(*Petaurus breviceps*)	Pollen, sap &invertebrates	Range of *Eucalyptus* forests	30 to 60 ha	Patchy distribution, Low densities(0.05 to 0.14 individuals per ha), saptrees, habitat loss, habitat degradation& large home ranges (30 to 60 ha)	Exudivore
Greater glider(*Petauroides volans*)	*Eucalyptus* leaves &flowers	Tall, wet *Eucalyptus* forests	1.3 to 2.5 ha	Tree hollows, habitat loss &modification	Folivore

1 Home-range in disturbed environment;

2 Home-range in forest environment;

3 Home-range of males;

4 Home-range of females. References used in the compiling of this table include [13], [14], [27], [30], [32]–[39].

### Determining Occurrences of Arboreal Marsupials

Tree cavities provide arboreal marsupials with critical den and breeding sites [40]. Two techniques are therefore useful for studying possums and gliders, snag-watching and spotlighting. Snag-watching is typically utilised to establish breeding and nesting sites for arboreal marsupials [41]. Observers identify, watch, and count the arboreal marsupials emerging from hollows in snag trees at dusk [41]. Spotlighting is a more commonly used sampling strategy for presence-only and presence absence studies that relate occurrence to habitat variables [41]. Spotlighting entails traversing a predetermined line transect by car or on foot, using a spotlight to identify arboreal marsupials [20].

We characterized spotlight transects based on several ecological variables using ArcGIS 10.0 [42] and undertook ground truthing prior to commencement. Tree and land cover estimates were used to allocate street transects as extreme urban, high urban and low urban (Table 2). Land and tree cover were also used to define remnant transects. Remnant patches were placed in the categories of small (5 to 15 ha), medium (15 to 30 ha) and large (30 to 45 ha). To ensure adequate coverage of the forested component of the gradient we established transects in the dominant ecological vegetation classes (e.g. wet, damp and riparian [43]) throughout the forest region. In total our sampling approach led to the establishment of 54 spotlight transects across the urban gradient. Within each of the street, remnant and forest components of the gradient we established 18 transects. In each transect type class we had six representative transects (Table 2). Due to spatial constraints forest transects were 1000 m while street and remnant transects were 500 m. As this research is modelling presences only, the difference in transect length does not influence the final models.

**Table 2:** Ecological characteristics used to define spotlight transects for arboreal marsupials.

Transect	Transect type	Characteristics	Transect length (m)	Number of transects	Number of transects
category				per category	per type
***Street***	Extreme Urban	Tree cover<scattered^1^	500 m		6
		Predominant land cover = impervious surfaces			
	High Urban	Tree cover scattered^2^	500 m	18	6
		Predominant land cover = impervious surfaces			
	Low Urban	Tree cover moderate^3^	500 m		6
		Predominant land cover = impervious surfaces			
***Remnant***	Small Remnant Large	Dense Tree Cover^4^	500 m		6
		Remnants between 5 and 15 ha			
	Medium Remnant	Dense Tree Cover^4^	500 m	18	6
		Remnants between 15 and 30 ha			
	Remnant	Dense Tree Cover^4^	500 m		6
		Remnants between 30 and 45 ha			
***Forest***	Wet Forest	Dense Tree cover^4^	1000 m		6
		>100 ha size Wet Forest EVC^5^			
	Damp Forest	Dense Tree cover^4^	1000 m	18	6
		>100 ha size Damp Forest EVC^6^			
	Riparian Forest	Dense Tree cover^4^	1000 m		6
		>100 ha size Riparian Forest EVC^7^			

1 Less than ‘Scattered’ tree cover represents <10% crown cover density, allowing for gaps of 0.1 ha;

2 ‘Scattered’ tree cover represents 10 to 50% crown cover density, allowing for gaps of 0.1 ha;

3 ‘Moderate’ tree cover represents 50–80% crown cover density, allowing for gaps of 0.25 ha;

4 ‘Dense’ tree cover represents >80% crown cover density, allowing for gaps in tree cover of up to 0.1 ha.

5 ‘Wet forest’ includes vegetation types where moisture is rarely a limiting factor and plants in these environments have little drought tolerance;

6 ‘Damp forest’ includes vegetation types where moisture is usually not a limiting factor but may become a factor in drought conditions, therefore plants in these environments have some adaptation to water stress;

7 ‘Riparian forest’ are areas of forest adjacent to a river or creek that require the presence of free water during the year either through average river flows or floods.

We surveyed each transect seven times between August 2007 and February 2008, but never in adverse weather, to ensure that the maximum number of presence locations for each species was determined. To reduce the occurrence of survey bias, transect spotlighting order was rotated per visit. In order to allow species time to leave diurnal dens we commenced spotlighting an hour after dusk, and continued up until an hour before dawn. We surveyed a maximum of eight transects per night to reduce observer fatigue and subsequent observer bias. Using a handheld spotlight all transects were surveyed on foot by BI and one volunteer.

We completed continuous forest transects in one hour, while urban and remnant transects were undertaken in 30 minutes due to their shorter length. The longest sighting distance of 50 m occurred in the disturbed remnants. We used both direct observation (e.g. size, shape and colouration) and indirect identification via eye shine (e.g. yellow to orange for common ringtail possums, orange to red for common brushtail possums, pale red for sugar and yellow-bellied gliders and white for greater gliders [44], [45]) to identify species. For each observation we recorded the species observed, position along the transect where the observation occurred, distance to the animal and the angle of observation from the transect. We used these last three measurements to generate specific spatial locations for each observation. Direct GPS locations could not be recorded as this would entail leaving the transect and potentially disturbing other individuals allowing them to escape prior to detection.

### Combining Field and Atlas Data

Museums, herbariums and government bodies have stored vast amounts of data detailing the occurrence of flora and fauna that can be used to supplement field collected data [46]. Presence data in Victoria is managed by the Department of Environment and Primary Industries (DEPI) within an electronic atlas referred to as the Atlas of Wildlife. In order to gain a representative distribution of presence locations for modelling we supplemented our data collected by spotlighting, with arboreal marsupial presence records from the aforementioned atlas. We corrected for known bias in the atlas records associated with incorrect observations and historical environmental change, by restricting records to those collected between 1997 and 2011 that were assessed as accurate by DEPI.

After we combined the presence data from the aforementioned sources, we removed duplicate presences by creating a point layer with a maximum of one presence point per 20 m×20 m. This removal of duplicate presence points was conducted to account for spatial auto-correlation. This substantially reduced the number of presence records in our study for several of the target species, resulting in arboreal marsupials being merged into broad groups for modelling purposes. We defined three groups of arboreal marsupials for modelling purposes based on their perceived susceptibility to disturbance. These groupings were referred to as disturbance-intolerant, tolerant to moderate disturbance and disturbance-tolerant. Disturbance-intolerant included presences for the greater glider, mountain brushtail possum and yellow-bellied glider [14]. Those tolerant of moderate disturbance included the sugar glider [47] and disturbance-tolerant included presences for the common ringtail and common brushtail possums [48]. These groupings were used in order to establish whether susceptibility to disturbance (e.g. urbanization) limited potential habitat across the gradient.

### Accounting for Sample Selection Bias

A limitation associated with presence-only data and presence-only models is sample selection bias [49]. We used atlas data from the DEPI Atlas of Wildlife to construct bias layers to overcome this issue. We collated all records of vertebrate nocturnal terrestrial species recorded across the study region between 1997 and 2011 from the atlas, and used 1/euclidean distance to all vertebrate nocturnal terrestrial presence points to form a bias layer. This technique rates cells across the landscape in relation to survey effort. Intensively surveyed cells receive a high bias rating while those with minimal survey effort receive low bias ratings allowing models to correct predictions based on survey effort.

### Eco-geographical Variables

Prior research on arboreal marsupials suggests that their tolerance to anthropogenic impacts is linked to patch metrics (e.g. patch size, connectivity), habitat structure, food and denning availability [14], [16], [19], [30]. We produced 11 eco-geographical variables (EGVs) for modelling purposes including: Euclidean distance to riparian vegetation, tree cover, a digital terrain model, riparian vegetation, normalised difference vegetation index (NDVI), land cover, slope position classification (SPC), lineal density of ephemeral rivers, permanent rivers, rivers and roads. We created the NDVI and the land cover layer using SPOT 5 satellite imagery (Supplementary 1). EGV’s produced for arboreal marsupial modelling had a spatial resolution of 20 m×20 m and were broadly classified as ecological, geographic and anthropogenic (Table 3).

**Table 3:** Original and derived eco-geographical variables for modelling.

Derived Layer/s	Variable Type	Data Type	Categories	Layer/Data Source
Lineal density of rivers	E	Continuous	–	Rivers - VICMAP (HYDRO25)
Lineal density of ephemeral rivers	E	Continuous	–	
Lineal density of Permanent rivers	E	Continuous	–	
Lineal density of roads	A	Continuous	–	Roads - VICMAP (VMTRANS)
Euclidean distance to riparian vegetation	E	Continuous	–	Ecological Vegetation Classes
Riparian vegetation	E	Categorical	Present	(EVC)NV2005_EVCBCS Department of
			Absent	Sustainability and Environment
Normalised Difference Vegetation Index	A,E	Continuous	–	
Land cover	A,E	Categorical	Impervious surfaces	SPOT 5 Imagery - SPOT5
			Tree Cover	(Systèm Pour l’Observation de la Terre)
			Grass/agriculture	
			Rivers	
			Waterbodies	
DTM20 m	G	Continuous	–	
Slope position classification (SPC)	G	Categorical	Ridge	Digital Terrain Model (DTM) 20 m - VICMAP
			Upper slope	
			Middle slope	
			Flat slope	
			Lower slope	
			Valley	
Tree cover density	E	Categorical	Dense (>80% crown cover density)	Tree Cover Density (percent cover) –VICMAP
			Moderate (50–80% crown cover density)	(TREEDEN25)
			Scattered (10–50% crown cover density)	
			None (<10% crown cover density)	

Variable type E equates to an ecological variable, Variable type A equates to an anthropogenic variable, Variable type G equates to a geographical variable.

### Model Building and Evaluation

Two main types of ecological models exist, those that require both presences and absences (e.g. presence/absence models) and those that rely solely on presences (e.g. presence-only models) with both having associated advantages and disadvantages [46]. Presence-only modelling is advantageous, because only presence data is required. We used presence only modelling to infer habitat suitability for arboreal marsupials across a gradient based on known presences. Presence-only modelling also places less importance on knowing the detection probabilities of species, as we do not have to assign absences to locations where no presences have been recorded. This becomes critical when using data sources such as atlas records.

Before modelling, we used ENM tools (version 1.3) [50] to determine correlation between the different EGVs. EGVs were considered highly correlated if R^2^≥0.75, in which case, we retained the most ecologically relevant variable for modelling [51]. Maxent [52], is a machine learning process, that determines the spatial probability distribution of a species, based on association between presences and EGVs then infers habitat suitability indices across the broader landscape, thus creating habitat suitability models [53]. We used Maxent to identify the association between presences and EGVs and in turn produced presence-only species distribution models for arboreal marsupials across a gradient of urbanization. We ran all models, using a composition of default settings and alternative settings, where ecologically applicable [54]. Twenty replications of each model were run, incorporating 5000 iterations. Random selection partitioned the data 75% to 25% per run, where 75% of the data were used to train the model and the remaining 25% for testing [54]. To gauge the effects of model complexity we ran models at regularisation β-multipliers of 0.5, 1, 2, 3, 4 and 5. We also ran all models with or without the bias layer, creating a total of 12 different final models for each arboreal marsupial group.

We ascertained model fit using the area under the receiver operator curve [55]. Models with the highest model fit, or AUC scores were then evaluated using ENM tools [50], [56]. ENM tools produce Akaike Information Criterion (AICc) scores for all models, the model with the lowest AICc score being the most parsimonious model. We transferred the best suitability model for each group of arboreal marsupial into ArcGIS to apply a binary threshold. Caution is suggested when using thresholds to classify presence only model outputs, unless conducting further analysis [54]. Logistic habitat suitability maps have a scale ranging from 0 to 100, where 0 equates to habitat being unsuitable for the species through to 100 or optimal habitat suitability. We required a binary map to conduct post proportional analysis, therefore a 10th percentile threshold was applied. This threshold is commonly used in SDM due to its conservative nature, which produces more ecologically applicable results [51], [57].

### Impact of Urbanization and Arboreal Marsupial Diversity across the Gradient

Proportional analysis for urban, urban-fringe and forest environments was completed in ArcGIS 10.0. Proportional analysis was used to establish the impact of the urbanization gradient on potential habitat for the three broad arboreal marsupial groups. A total of 75 1 km×1 km (area = 100 ha) sites was randomly established, 25 in each zone along the gradient. In each sample site the amount of potential habitat was determined for each of the three arboreal marsupial groups, to evaluate response to zones along the gradient. Diversity across the urbanization gradient was derived using potential habitat suitability and the number of functional arboreal marsupial groups present.

ANOVAs were completed in IBM SPSS statistics 20.0 [58] to examine whether a difference occurred in the availability of potential habitat for each of the three groups of arboreal marsupials across the gradient. Diversity of arboreal marsupials and assemblage changes were assessed by comparing the amount of potential habitat for all three groups, two of the three groups, one of the three groups and none of the three groups across the zones of the gradient. A significance level of 0.05 was used. Tukeys’ post-hoc test was used to identify homogenous subsets.

## Results

### Species Presence Data

We collected a total of 2740 arboreal marsupial records, 870 from spotlight surveys and 1870 from the DEPI Atlas of Wildlife. The number of records per species varied, with more presence records for the conspicuous species (e.g. mountain brushtail possums, common ringtail and common brushtail possums). Correcting for autocorrelation substantially reduced presence records for all species (Table 4). After combining species records into groups, and recorrecting for spatial autocorrelation we retained 306 presence records for the disturbance-intolerant group (e.g. greater glider, yellow bellied glider and mountain brushtail possum), 134 presences for the group with moderate tolerance (e.g. sugar gliders) and 637 presence records for the disturbance-tolerant group (e.g. common ringtail and common brushtail possums), which were used for modelling purposes.

**Table 4:** Collection and refinement of presence-only data.

	GG	MBP	YBG	SG	CRP	CBP	Total
**Initial presences**	205 *(40)*	359 *(163)*	318 *(50)*	239 *(27)*	617 *(209)*	1002 *(417)*	2740 *(906)*
**Presences (duplicates removed)**	87	161	119	133	330	412	1242
	**Disturbance-intolerant**			**TMD**	**Disturbance-tolerant**		**Total**
**Presences when merged into groups**	367			133	742		1242
**Presences when merged into groups and duplicates removed**	306**			133**	637**		1077

**GG** stands for greater glider; **MBP** stands for mountain brushtail possum; **YBG** stands for yellow-bellied glider; **SG** stands for sugar glider; **CRP** stands for common ringtail possum; **CBP** stands for common brushtail possum. Normal text indicates total records per species while bracketed italic text indicates how many of these records were collected in the present study. **TMD** stands for tolerance of moderate disturbance. Numbers of final presences used in models are those represented by **.

### Habitat Suitability Models and Evaluation

We performed correlation analysis, prior to modelling, which indicated that several of the EGV’s were highly correlated (R^2^≥0.75). Lineal density of rivers were highly correlated with lineal density of roads (R^2^ = 0.75). Lineal density of ephemeral rivers were also highly correlated with the lineal density of roads (R^2^ = 0.75) and lineal density of rivers (R^2^ = 1.00) (Table S1). In relation to the correlated variables, each model used a different combination of variables, with perceived tolerance of the arboreal marsupial group to disturbance as the determinant of which correlated variables were retained.

We produced a total of 36 models, 12 models for each of the arboreal marsupial groups (e.g. disturbance-intolerant, moderate tolerance to disturbance and disturbance-tolerant). AUCtest scores across the 36 models ranged from 0.75 to 0.91. The most parsimonious model for each arboreal marsupial group had the highest AUC, lowest AICc, no bias layer and a variable beta-multiplier (Table 5). We established that the amount of potential habitat for disturbance-intolerant species and those with moderate tolerance to disturbance varied in response to the urbanization gradient (Disturbance-intolerant: F_2,72_ = 156.84, p<0.001; Moderate tolerance to disturbance: F_2,72_ = 418.67, p<0.001). Amount of potential habitat for disturbance-tolerant species was distributed evenly across the gradient (F_2_,_72_ = 18.21, p = 0.050).

**Table 5:** Final model parameters.

	Disturbance-intolerant	Moderate tolerance to	Disturbance-tolerant
	Models EVG’s	disturbance Models EVG’s	Models EVG’s
	Tree cover, Linden eph rivers, Lindenperm rivers, Land cover, NDVI,Riparian, DTM, SPC, Eucdistriparian veg	Tree cover, Linden eph rivers, Lindenperm rivers, Land cover, NDVI,Riparian, DTM, SPC, Eucdistriparian veg	Tree cover, Linden perm rivers,Land cover, NDVI, Linden roads,Riparian, DTM, SPC, Eucdistriparian veg
**Model 1**	*Reg β-multi* = 0.5	*Reg β-multi* = 0.5	*Reg β-multi* = 0.5***
	*Bias Layer* = No Bias Layer	*Bias Layer* = No Bias Layer	*Bias Layer* = No Bias Layer
**Model 2**	*Reg β-multi* = 1***	*Reg β-multi* = 1	*Reg β-multi* = 1
	*Bias Layer* = No Bias Layer	*Bias Layer* = No Bias Layer	*Bias Layer* = No Bias Layer
**Model 3**	*Reg β-multi* = 2	*Reg β-multi* = 2***	*Reg β-multi* = 2
	*Bias Layer* = No Bias Layer	*Bias Layer* = No Bias Layer	*Bias Layer* = No Bias Layer
**Model 4**	*Reg β-multi* = 3	*Reg β-multi* = 3	*Reg β-multi* = 3
	*Bias Layer* = No Bias Layer	*Bias Layer* = No Bias Layer	*Bias Layer* = No Bias Layer
**Model 5**	*Reg β-multi* = 4	*Reg β-multi* = 4	*Reg β-multi* = 4
	*Bias Layer* = No Bias Layer	*Bias Layer* = No Bias Layer	*Bias Layer* = No Bias Layer
**Model 6**	*Reg β-multi* = 5	*Reg β-multi* = 5	*Reg β-multi* = 5
	*Bias Layer* = No Bias Layer	*Bias Layer* = No Bias Layer	*Bias Layer* = No Bias Layer
**Model 7**	*Reg β-multi* = 0.5	*Reg β-multi* = 0.5	*Reg β-multi* = 0.5
	*Bias Layer* = Bias Layer	*Bias Layer* = Bias Layer	*Bias Layer* = Bias Layer
**Model 8**	*Reg β-multi* = 1	*Reg β-multi* = 1	*Reg β-multi* = 1
	*Bias Layer* = Bias Layer	*Bias Layer* = Bias Layer	*Bias Layer* = Bias Layer
**Model 9**	*Reg β-multi* = 2	*Reg β-multi* = 2	*Reg β-multi* = 2
	*Bias Layer* = Bias Layer	*Bias Layer* = Bias Layer	*Bias Layer* = Bias Layer
**Model 10**	*Reg β-multi* = 3	*Reg β-multi* = 3	*Reg β-multi* = 3
	*Bias Layer* = Bias Layer	*Bias Layer* = Bias Layer	*Bias Layer* = Bias Layer
**Model 11**	*Reg β-multi* = 4	*Reg β-multi* = 4	*Reg β-multi* = 4
	*Bias Layer* = Bias Layer	*Bias Layer* = Bias Layer	*Bias Layer* = Bias Layer
**Model 12**	*Reg β-multi* = 5	*Reg β-multi* = 5	*Reg β-multi* = 5
	*Bias Layer* = Bias Layer	*Bias Layer* = Bias Layer	*Bias Layer* = Bias Layer
**AUCtest** **score range**	0.89 to 0.91	0.75 to 0.76	0.75 to 0.81
**Parameters** **of the most** **parsimonious** **model**	*AUCtrain = *0.932	*AUCtrain = *0.826	*AUCtrain = *0.861
	*AUCtest = *0.910	*AUCtest = *0.76	*AUCtest = *0.813
	*Bias layer = *No	*Bias layer = *No	*Bias layer = *No
	*Reg β-multi = *1	*Reg β-multi = *2	*Reg β-multi = *0.5

**Linden eph rivers** equates to Lineal density of ephemeral rivers; **Linden perm rivers** equates to Lineal density of permanent rivers; **NDVI** equates to Normalised Difference Vegetation Index; **DTM** equates to Digital Terrain Model; **SPC** equates to Slope Position Classification, **Eucdist riparian veg** equates to Euclidean distance to riparian vegetation; **Linden roads** equates to Lineal density of roads. Reg β-multi equates to regulation β-multiplier (smoothing parameter). ***Indicates best models for Disturbance sensitive, Disturbance Tolerant and Generalist/Opportunistic Arboreal marsupials.

### Disturbance-intolerant Species

From the nine EGV’s used to construct the most parsimonious model, lineal density of ephemeral rivers, NDVI and Euclidean distance to riparian vegetation, accounted for 64.9% of the model performance. Despite contributing only 13.9% to the model, model outputs suggested that tree cover was the most important variable if used in isolation. Model outputs also indicated that lineal density of ephemeral rivers contributed the most unique information to the model at 30.2%. We established that potential habitat for disturbance-intolerant arboreal marsupials occurred in conjunction with tree canopy cover exceeding 80% and higher values in the NDVI greenness index (an indication of plant biomass) (Figure S1a,b).

Potential habitat was also influenced by increased lineal densities of ephemeral rivers, but declined once lineal density of ephemeral rivers reached three lineal km per km^2^ (Figure S1c). Increases in lineal density of permanent rivers had the same effect on potential habitat as lineal density of ephemeral rivers. Distance from riparian vegetation was also a driver of potential habitat for disturbance-intolerant species, with occurrence of potential habitat declining sharply at ≥3 km from riparian vegetation (Figure S1d). This group was also more likely to occur at higher elevations.

**Figure 2:**
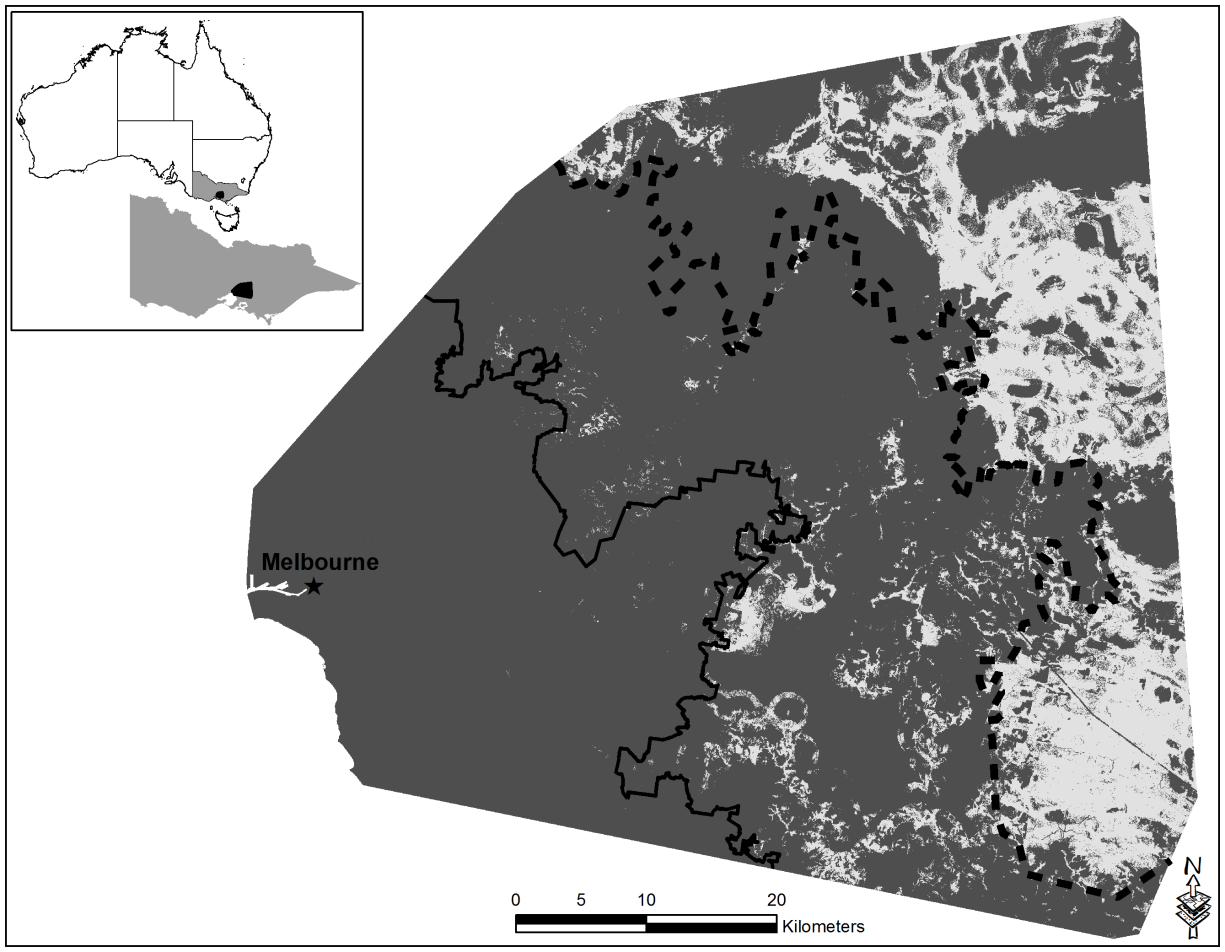
Habitat suitability map for disturbance-intolerant arboreal marsupials over an urbanization gradient in south eastern Australia based on Maxent model predictions. Lighter areas represent potential habitat and dark grey no potential habitat. The constant black line represents the urban to urban-fringe boundary, while the dashed black line highlights the urban-fringe to forest boundary.

The amount of potential habitat for disturbance-intolerant arboreal marsupial species varied across the urban gradient (F_2,72_ = 156.84, p<0.001). Potential habitat for disturbance-intolerant species was highest in the forest zone (70.5%) (Tukey p<0.05), with the urban-fringe providing limited potential habitat (11.4%) and the urban zone providing no potential habitat (0%) (Tukey p<0.05) (Figure 2).

### Species with Moderate Tolerance to Disturbance

Tree cover, land cover and lineal density of ephemeral rivers contributed 69.9% of the model performance for species with moderate disturbance tolerance. Model outputs indicated that tree cover was the most important contributing variable at 32.2% and also contributed the most unique information to the model. We ascertained that potential habitat for disturbance-tolerant arboreal marsupials increased where tree crown cover exceeded 80% and where adequate water sources occurred (Figure S2a,b). Increases in the lineal density of ephemeral rivers resulted in an increase in the probability of potential habitat for tolerant species (Figure S2c).

**Figure 3:**
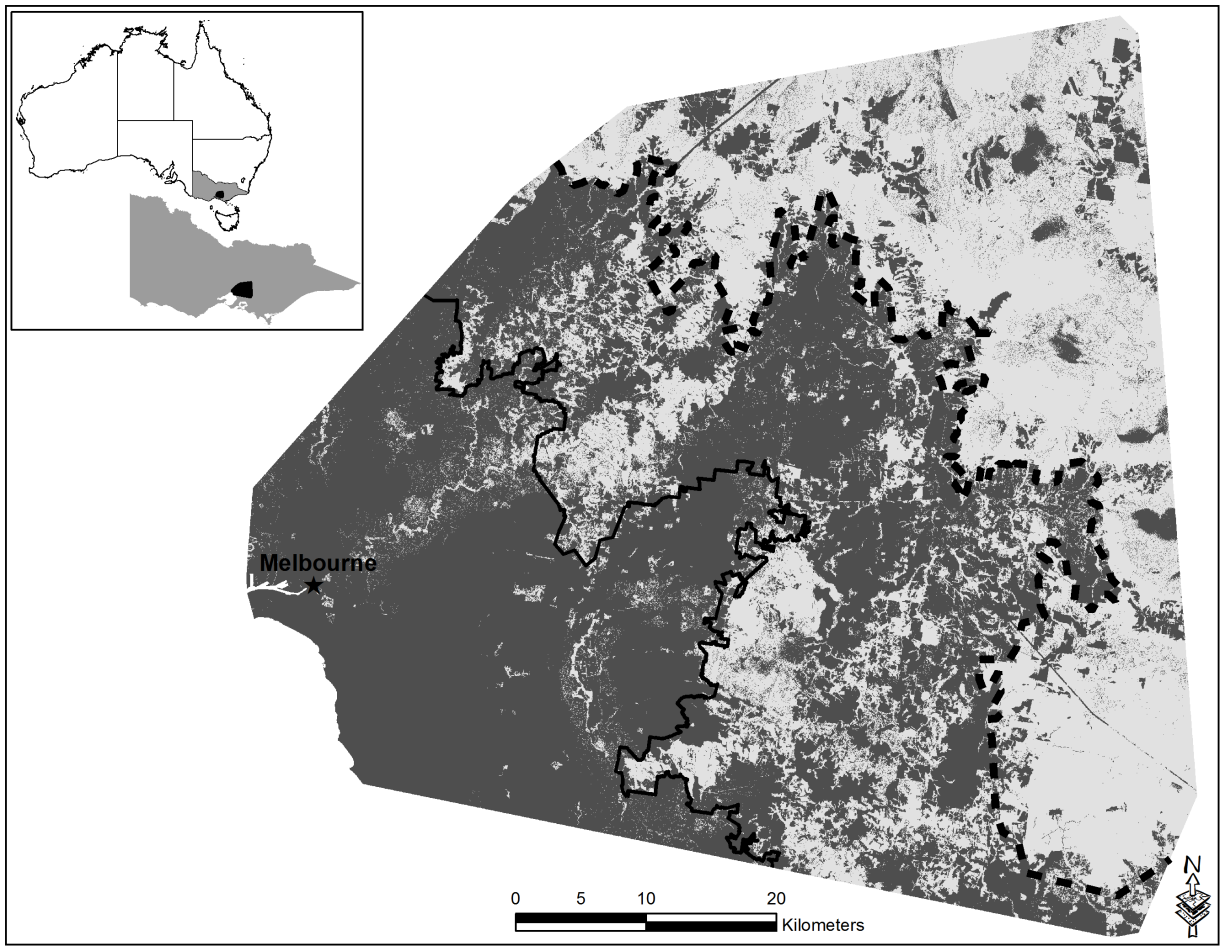
Habitat suitability map for arboreal marsupials with moderate tolerance to disturbance over an urbanization gradient in south eastern Australia based on Maxent model predictions. Lighter areas represent potential habitat and dark grey no potential habitat. The constant black line represents the urban to urban-fringe boundary, while the dashed black line highlights the urban-fringe to forest boundary.

Lineal density of permanent water sources, also influenced potential habitat, but permanent river densities of greater than 1.1 lineal kilometers per km^2^ caused a decline in the probability of encountering potential habitat. Euclidean distance to riparian vegetation was also an important EGV with the occurrence of potential habitat declining with distance from riparian vegetation. Potential habitat for species with a moderate disturbance tolerance also increased in line with elevation, and peaked at elevations greater than 200 m. The amount of potential habitat for species with moderate disturbance tolerance differed across the gradient (F_2,72_ = 418.67, p<0.001), being highest in the forest (76.4%), moderate the urban fringe at 47.9% (Tukey p<0.05), and lowest in urban environments (4.9%) (Tukey p<0.05) (Figure 3).

### Disturbance-tolerant Species

**Figure 4:**
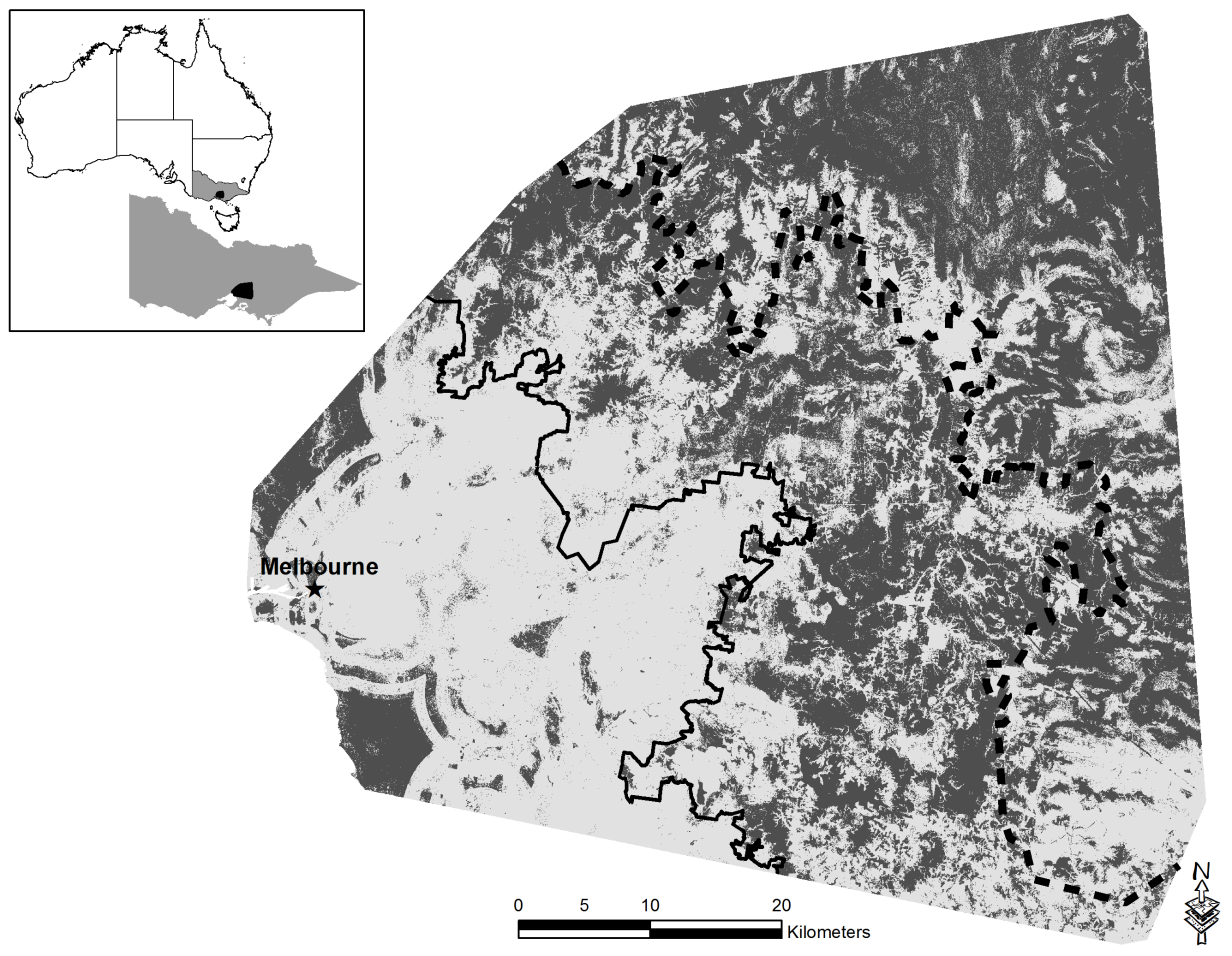
Habitat suitability map for disturbance-tolerant arboreal marsupials over an urbanization gradient in south eastern Australia based on Maxent model predictions. Lighter areas represent potential habitat and dark grey no potential habitat. The constant black line represents the urban to urban-fringe boundary, while the dashed black line highlights the urban-fringe to forest boundary.

The DTM and lineal density of roads contributed 69.9% of the model performance. Lineal density of roads was the most important contributing variable at 23%, in addition to being the variable contributing the most unique information. Potential habitat for disturbance tolerant species was associated with lineal density of roads, increasing to a peak occurrence at 7.5 lineal km of roads per km^2^ (Figure S3a). We determined that increasing elevation (Figure S3b) and Euclidean distances of 6 km or greater away from riparian vegetation caused declines in the occurrence of potential habitat for disturbance-tolerant species. The amount of potential habitat for disturbance-tolerant species was similar across the gradient, with urban zones providing similar amounts of potential habitat to the urban-fringe and forest (F_2_,_72_ = 18.21, p = 0.050)(urban: 68.2%, urban-fringe: 54.0%, forest 51.1%) (Figure 4).

### Arboreal Marsupial Assemblages across the Urban Gradient

**Figure 5:**
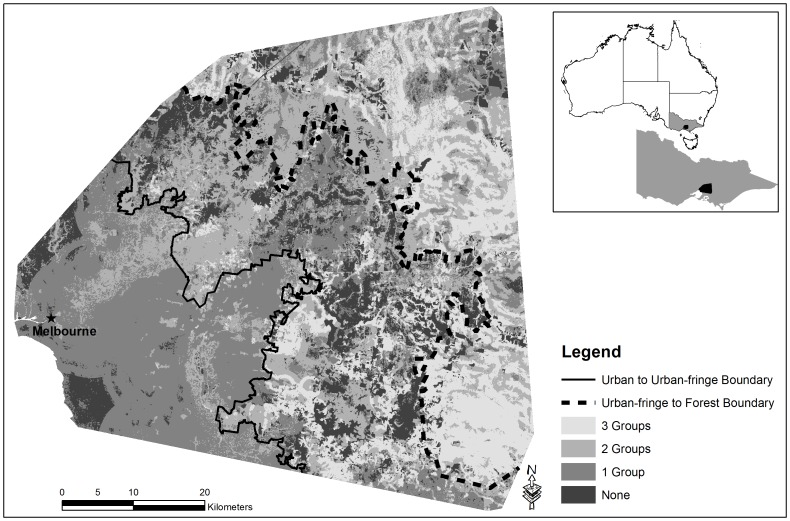
Habitat suitability map indicating the number of arboreal marsupials groups across the urbanization gradient.

We constructed a composite map of areas supporting potential habitat for all three groups of arboreal marsupials (diverse assemblages), two of the three groups (moderate diversity in assemblages), one of the three groups (low diversity in assemblages) and none of the three groups. This established that diversity declined in arboreal marsupial assemblages, in line with increasing urbanization (Diverse assemblages: F_2,72_ = 52.21, p<0.001, Moderate diversity in assemblages: F_2,72_ = 44.62, p<0.001, Low diversity in assemblages: F_2,72_ = 57.49, p<0.001, none of the disturbance groups: F_2,72_ = 8.33, p = 0.001) (Figure 5).

**Figure 6:**
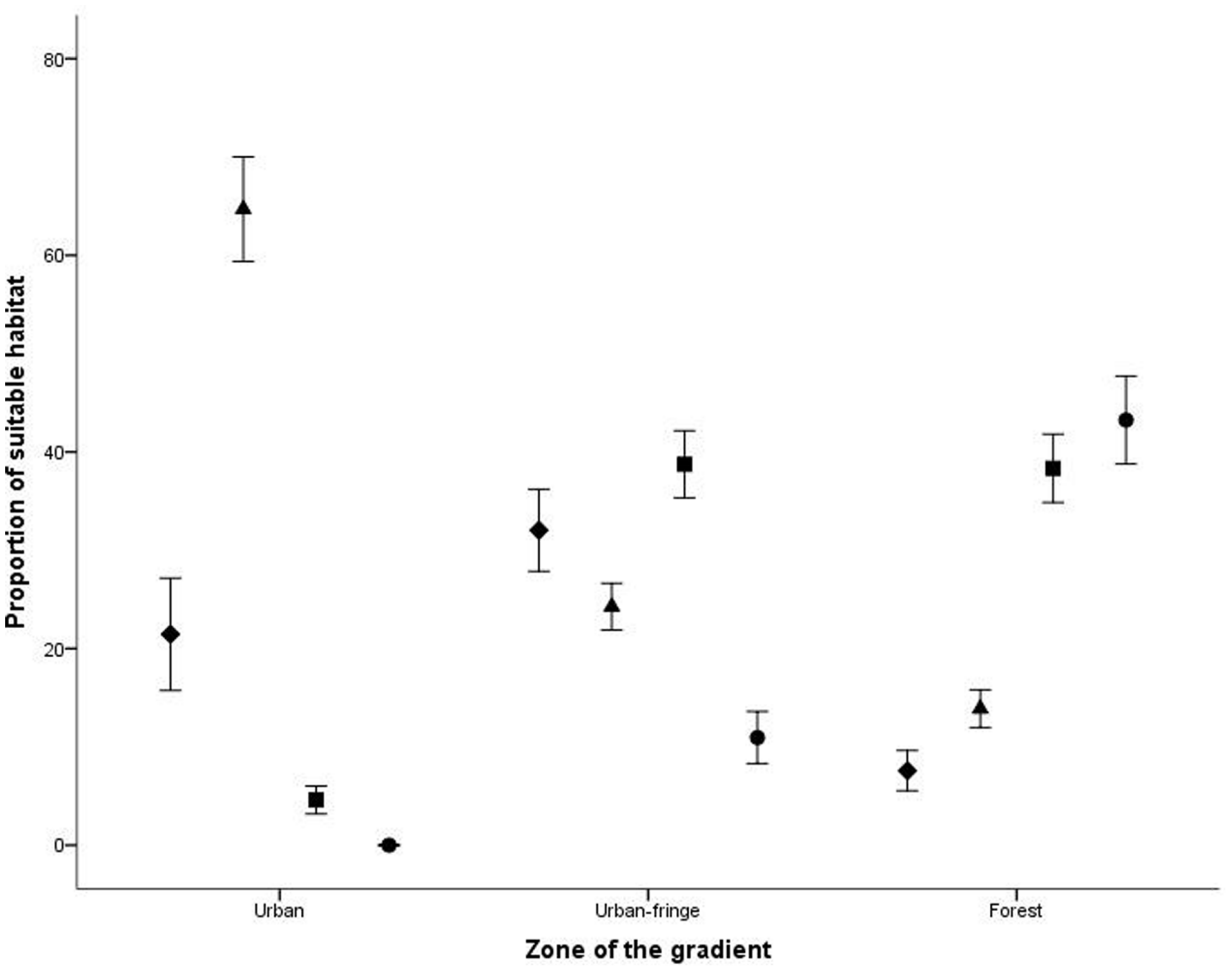
Proportion of potential habitat and diversity of arboreal marsupial groups (±1 SE) in urban, urban-fringe and forest zones where diamonds represent no disturbance groups, triangles represent habitat suitability for one disturbance group, squares represent habitat suitability for two disturbance groups and circles represent habitat suitability for all three disturbance groups.

We determined that forest environments supported a greater amount of potential habitat for diverse arboreal marsupial assemblages (43.2%), which declined steadily with an increase in urbanization (urban: 0%) (Tukey<0.05) (Figure 6). The amount of potential habitat for moderately diverse arboreal marsupial assemblages was highest in the forest at 38.7% and urban fringe at 38.3% (Tukey>0.05), and limited in the urban zone (4.6%) (Tukey<0.05). Highly urbanized environments supported the highest amount of potential habitat for low diversity arboreal marsupial assemblages (urban: 64.7%) (Tukey<0.05), which declined in line with decreasing urbanization (Urban-fringe: 24.3%; Forest: 13.9%) (Tukey>0.05). We determined that the amount of potential habitat where none of the three disturbance groups were identified was highest in the urban-fringe and lowest in the forest (urban-fringe: 32.0%, forest: 7.6%) (Tukey<0.05), with urban zones containing intermediate amounts (21.5%) (Tukey>0.05).

## Discussion

Our research contributes to the understanding of urbanization processes across a gradient, demonstrating the linkages between spatial configurations of EGVs, potential habitat availability for arboreal marsupials and the simplification of arboreal marsupial assemblages with increasing urbanization. Alteration of flora and faunal communities in response to urbanization has been evidenced in Europe [7], [59], Africa [60], [61], North America [62], [63] and Australia [64], [65]. We questioned whether potential habitat for arboreal marsupials varied spatially in response to urbanization, and whether urbanization had the potential to simplify arboreal marsupial assemblages. Specifically, we found that potential habitat for arboreal marsupials did vary across the gradient, in line with species’ tolerance to disturbance and landscape composition. This suggests that urbanization is a precursor for the simplification of arboreal marsupial assemblages.

In our study area, the amount of potential habitat and diverse assemblages of arboreal marsupials were linked directly with forests, more specifically with natural tree cover, land cover, rivers and the proximity to riparian vegetation. Within the urban component of the gradient less diverse arboreal marsupial assemblages were evident and driven by anthropogenic variables, such as road density. Regardless of their position along the gradient riparian habitats proved important in predicting potential habitat for arboreal marsupials. This demonstrates that even in the most urbanized environments, intact riparian habitats may act as refuges for displaced species, as evidenced in avian research [66], [67].

The forest provided the greatest amounts of habitat for both disturbance-intolerant species and those with moderate disturbance tolerance. Potential habitat decreased substantially for disturbance-intolerant species within the urban-fringe, comprising of large forest remnants with high connectivity. A consequence of increasing urbanization is the reduction, simplification and isolation of patches of remnant vegetation [4], which appears to be detrimental to disturbance-intolerant species, such as the greater glider. This is likely linked to their specialist diets, large spatial requirements and the continuous canopy cover required for movement [16], [17]. As gaps in the canopy increase in width, in line with urbanization, these species are unable to maintain populations above a required threshold [68].

The sugar glider, with moderate tolerance to disturbance was able to persist across the gradient but as urbanization intensified the potential habitat for the sugar glider declined. Within the urban zone potential habitat for this species was limited to riparian vegetation following major river systems and larger isolated patches (Figure 3). This may be a response to the loss of complex floristic and structural resources for denning, nesting and foraging, as these resources are greatly reduced in the urban zone [17], [22], [47] or, more likely, a response to increased urbanization of the matrix [47].

Our hypothesis that moderate levels of disturbance would be detrimental to disturbance-intolerant species and advantageous to species with moderate tolerance to disturbance, before increases breached even their tolerance levels explained the patterns occurring in arboreal marsupial assemblages to a point. Disturbance-tolerant arboreal marsupials, the common ringtail and common brushtail possums did not conform to this hypothesis because potential habitat for these species occurred across the urbanization gradient and was available in similar amounts. While these species of arboreal marsupials may be affected by increased distance between remnant patches (e.g. collisions with cars) the provision of alternative food and nesting sources (e.g. ornamental gardens and buildings) allow populations of these species to reach higher densities in urbanized environments [20], [48].

Our research therefore suggests landscape composition is important for complex arboreal marsupial assemblages not only in terms of configuration but also the resources (e.g. food and dens) provided. The alteration of available resources by increasing urbanization processes is simplifying arboreal marsupial assemblages with diverse assemblages in forest environments reduced to two common species in highly urbanized environments. Simplification of faunal assemblages is detrimental, reducing resilience of faunal communities to change [69], [70], also altering trophic interactions [71], and ecosystem services [72].

Significant trophic implications arise from the simplification of arboreal marsupial assemblages. Arboreal marsupial assemblages in forest and urban-fringe environments contain both folivores and exudivores (Table 1). Folivore and exudivore dietary niches provide pollination and fertilization services to *Acacia* and *Eucalyptus* species [33]. Simplified assemblages within highly urbanized environments are reduced to folivores, that also exploit ornamental floral species. Any ecosystem services provided by folivores and exudivores in complex assemblages, but not by those in simplified assemblages, will be lost potentially further degrading urban vegetation.

Arboreal marsupials also provide bottom-up resources for predators. Arboreal marsupials are the primary prey for powerful owls [73]. Simplification of arboreal marsupial assemblages is unlikely to have an immediate negative affect on the powerful owl, as this species is an opportunistic predator, with diet composition variable both spatially and temporally [73], [74]. Long-term the simplification of arboreal marsupial assemblages could however, be considered detrimental to the powerful owl, with the removal of functional redundancies. If prey richness for this raptor is reduced to two species, the further loss of one or both of these prey sources, could potentially cause irreparable changes throughout upper trophic levels.

We acknowledge that across the urban to forest gradient, there may be other underlying climatic and geological gradients that would have initially influenced arboreal marsupial distributions. Recent research [20] and historical presence data (DEPI Wildlife Atlas) suggest that prior to European settlement and intensification of urbanization, many of these forest species (e.g. greater gliders, yellow-bellied gliders and mountain brushtail possums) would have maintained viable populations within forested and urban-fringe environments, which over time contracted due to habitat loss. This further attests to our hypothesis that simplification of arboreal marsupials is occurring across an urbanization gradient.

Continual increases in the world’s population, along with demand for housing and food production will continue to impact on biodiversity [1], however this must be balanced with the need to sustain complex flora and fauna communities, because simplification of biological communities has far reaching consequences.

## Supporting Information

Figure S1 **Response curves for disturbance-intolerant species.** Where a. equates to the tree cover response curve; b. equates to the NDVI response curve; c. equates to the lineal density of ephemeral rivers response curve; d. equates to the Euclidean distance to riparian vegetation response curve. Red represents the mean response of the variable over the 20 replicate runs in Maxent. Blue represents the mean response of the variable over the 20 replicate runs ± one standard deviation (Categorical variables contain two shades, blue and blue/green).
(TIF)

Figure S2 **Response curves for species with moderate tolerance to disturbance.** Where a. equates to the tree cover response curve; b. equates to the land cover response curve; c. equates to the lineal density of ephemeral rivers response curve. Red represents the mean response of the variable over the 20 replicate runs in Maxent. Blue represents the mean response of the variable over the 20 replicate runs ± one standard deviation (Categorical variables contain two shades, blue and blue/green).
(TIF)

Figure S3 **Response curves for disturbance-tolerant species.** Where a. equates to the lineal density of roads response curve; b. equates to the DTM response curve; Red represents the mean response of the variable over the 20 replicate runs in Maxent. Blue represents the mean response of the variable over the 20 replicate runs ± one standard deviation.
(TIF)

Table S1 **Correlation analysis of the 11 EGV’s conducted in ENM tools.**
(DOCX)

Supplementary S1 **Normalised Difference Vegetation Index (NDVI) production.**
(DOCX)

## References

[1] McKinney ML (2002) Urbanization, biodiversity, and conservation. BioSci 52: 883–890.

[2] McKinney ML (2006) Urbanization as a major cause of biotic homogenization. Biol Conserv 127: 247–260.

[3] Magle SB, Hunt VM, Vernon M, Crooks KR (2012) Urban wildlife research: Past, present, and future. Biol Conserv 155: 23–32.

[4] McDonnell MJ, Hahs AK, Breuste JH (2009) Ecology of cities and towns. Australia: Cambridge University Press. 746 p.

[5] Chace JF, Walsh JJ (2006) Urban effects on native avifauna: A review. Landsc Urban Plan 74: 46–69.

[6] Kark S, Iwaniuk A, Schalimtzek A, Banker E (2007) Living in the city: Can anyone become an ‘urban exploiter’? J Biogeogr 34: 638–651.

[7] Kühn I, Klotz S (2006) Urbanization and homogenization – Comparing the floras of urban and rural areas in Germany. Biol Conserv 127: 292–300.

[8] Melles S, Glenn SM, Martin K (2003) Urban bird diversity and landscape complexity: Species–environment associations along a multiscale habitat gradient. Ecol Society, 7, 5 Available: http://www.consecol.org/vol7/iss1/art5/. Accessed: 20 January 2010.

[9] Olden JD, Rooney TP (2006) On defining and quantifying biotic homogenization. Global Ecol Biogeogr 15: 113–120.

[10] Robertson OJ, McAlpine C, House A, Maron M 2013. Influence of interspecific competition and landscape structure on spatial homogenization of avian assemblages. PLoS ONE 8. DOI: 10.1371/journal.pone.0065299. 10.1371/journal.pone.0065299 PMC3665551 23724136

[11] Beyer GL, Goldingay RL (2006) The value of nest boxes in the research and management of Australian hollow-using arboreal marsupials. Wildl Res 33: 161–174.

[12] Lavazanian E, Wallis R, Webster A (1994) Diet of powerful owls (*Ninox strenua*) living near Melbourne, Victoria. Wildl Res 21: 643–645.

[13] Strahan R., van Dyck, S (2008) The mammals of Australia. Sydney, Australia: New Holland. 888 p.

[14] van der Ree R, Ward SJ, Handasyde KA (2004) Distribution and conservation status of possums and gliders in Victoria. In: Goldingay RL, Jackson SM, editors. The Biology of Australian Possums and Gliders. New South Wales: Surrey Beatty and Sons. pp. 91–110.

[15] Lindenmayer DB, Cunningham RB, Pope ML, Gibbons P, Donnelly CF (2000) Cavity sizes and types in Australian eucalypts from wet and dry forest types–a simple of rule of thumb for estimating size and number of cavities. For Ecol Manage 137: 139–150.

[16] Eyre TJ (2006) Regional habitat selection of large gliding possums at forest stand and landscape scales in southern Queensland, Australia: I. Greater glider (*Petauroides volans*). For Ecol Manage 235: 270–282.

[17] Wormington KR, Lamb D, McCallum HI, Moloney DJ (2002) Habitat requirements for the conservation of arboreal marsupials in dry sclerophyll forests of Southeast Queensland, Australia. Forest Sci 48: 217–227.

[18] Goldingay RL, Carthew SM, Funnell DL (1999) Feeding behaviour of the yellow-bellied glider (*Petaurus australis*) at the western edge of its range. Wildl Res 26: 199–208.

[19] Nelson JL, Cherry KA, Porter KW (1996) The effect of edges on the distribution of arboreal marsupials in the ash forests of the Victorian Central Highlands. Aust For 59: 189–198.

[20] Menkhorst PW, Loyn RH (2011) The mammalian fauna of Greater Melbourne: Diversity, loss, adaptation and change. The Vic Nat 128: 233–248.

[21] Suckling GC (1984) Population ecology of the sugar glider, *Petaurus breviceps*, in a system of fragmented habitats. Wildl Res 11: 49–75.

[22] Goldingay RL (2012) Characteristics of tree hollows used by Australian arboreal and scansorial mammals. Aust J Zool 59: 277–294.

[23] Kerle JA (2001) Possums: The brushtails, ringtails and greater glider. Sydney: University of New South Wales Press. 156 p.

[24] How RA, Kerle JA (1995) Common brushtail possum, *Trichosurus vulpecular*. In: Strahan R. editor. The mammals of Australia. Australia: Reed Books. pp. 273–275.

[25] Harper MJ, McCarthy MA, van Der Ree R (2008) Resources at the landscape scale influence possum abundance. Austral Ecol 33: 243–252.

[26] Pahl L (1984) Diet preference, diet composition and population density of the ringtail possum (*Pseudocheirus peregrinus cooki*). In: Smith AP, Hume ID, editors. Possums and Gliders. Sydney, Australia: Australian Mammal Society and Beatty and Sons. pp. 252–260.

[27] Harper MJ (2005) Home range and den use of common brushtail possums (*Trichosurus vulpecula*) in urban forest remnants. Wildl Res 32: 681–687.

[28] Soderquist TR, Mac Nally R (2000) The conservation value of mesic gullies in dry forest landscapes: Mammal populations in the box–ironbark ecosystem of southern Australia. Biol Conserv 93: 281–291.

[29] Lindenmayer DB, Cunningham RB, Donnelly CF (1997) Decay and collapse of trees with hollows in eastern Australian forests: Impacts on arboreal marsupials. Ecol Appl 7: 625–641.

[30] Kavanagh RP, Wheeler RJ (2004) Home-range of the greater glider *Petauroides volans* in tall montane forest of southeastern New South Wales, and changes following logging. In: Goldingay RL, Jackson SM, editors. The Biology of Australian Possums and Gliders. New South Wales: Surrey Beatty and Sons. pp. 413–425.

[31] Brenden T, Wang L, Su Z (2008) Quantitative identification of disturbance thresholds in support of aquatic resource management. Environ Manage 42: 821–832. 18491181 10.1007/s00267-008-9150-2

[32] Goldingay RL, Sharpe DJ (2004) Spotlights versus nextboxes for detecting feathertail gliders in north-east New South Wales. In: Goldingay RL, Jackson SM, editors. The Biology of Australian Possums and Gliders. New South Wales: Surrey Beatty and Sons. pp. 298–305.

[33] Goldingay RL, Jackson SM (2004) A review of the ecology Petauridae. In: Goldingay RL, Jackson SM, editors. The Biology of Australian Possums and Gliders. New South Wales: Surrey Beatty and Sons. pp. 376–400.

[34] Martin JK (2006) Den-use and home-range characteristics of bobucks, *Trichosurus cunninghami*, resident in a forest patch. Aust J Zool 54: 225–234.

[35] Broom LS (2001) Density, home range, seasonal movements and habitat use of the mountain pygmy-possum *Burramys parvus* (Marsupialia: Burramyidae) at Mount Blue Cow, Kosciuszko National Park. Austral Ecol 26 275–292: 36.

[36] Statham M, Statham HL (1997) Movements and Habits of Brushtail Possums (*Trichosurus vulpecular* Kerr) in an Urban Area. Wildl Res 24: 715–726.

[37] Haris JM (2008) *Cercartetus nanus* (Diprotodontia: Burramyidae). Mamm Species 815: 1–10.

[38] Lindenmayer D (1996) Wildlife and woodchips: Leadbeater’s possum: a test case for sustainable forestry. Sydney: UNSW Press. 156p.

[39] Comport SS, Ward SJ, Foley WJ (1996) Home ranges, time budgets and food-tree use in a high-density tropical population of greater gliders, *Petauroides volans* (Pseudocheiridae: Marsupialia). Wildl Res 23: 401–419.

[40] Gibbons P, Lindenmayer DB (2002) Tree hollows and wildlife conservation in Australia. Collingwood, Australia: CSIRO Publishing. 240 p.

[41] Smith AP, Lindenmayer D, Begg RJ, Macfarlane MA, Seebeck JH, Suckling GC (1989) Evaluation of the stag-watching technique for census of possums and gliders in tall open forest. Australian Wildl Res 16: 575–580.

[42] Environmental Systems Reasearch Institute (2010) ArcGIS 10.0. Redlands, CA: Environmental Systems Research Institute.

[43] Department of Environment and Primary Industries (DEPI) (2013) Ecological Vegetation Class (EVC) Benchmarks for each Bioregion. Melbourne, Australia: Department of Environment and Primary Industries. Available: URL: http://www.dse.vic.gov.au/conservation-and-environment/ecological-vegetation-class-evc-benchmarks-by-bioregion. Accessed: 10 June 2012.

[44] Smith A, Winter J (1997) A key and field guide to the Australian possums, gliders and koala. New South Wales, Australia: Surrey Beatty and Sons. 52p.

[45] Lindenmayer D (2002) Gliders of Australia: A natural history. Sydney, New South Wales: UNSW Press. 160p.

[46] Elith J, Phillips SJ, Hastie T, Dudík M, Chee YE, Yates CJ (2011) A statistical explanation of MaxEnt for ecologists. Divers Distrib 17: 43–57.

[47] Caryl FM, Thomson K, van der Ree R (2013) Permeability of the urban matrix to arboreal gliding mammals: Sugar gliders in Melbourne, Australia. Aust Ecol 38: 609–616.

[48] Temby ID (2004) Urban wildlife issues in Australia. In: Shaw WW, Harris LK, VanDruff L, editors. Proceedings of the 4th International Symposium on Wildlife Conservation. Tuscon, Arizona: The University of Arizona. pp. 26–34.

[49] Phillips SJ, Dudík M, Elith J, Graham CH, Lehmann A, Leathwick J, Ferrier S (2009) Sample selection bias and presence-only distribution models: Implications for background and pseudo-absence data. Ecol Appl 19: 181–197. 19323182 10.1890/07-2153.1

[50] Warren DL, Glor RE, Turelli M (2010) ENMTools: A toolbox for comparative studies of environmental niche models. Ecography 33: 607–611.

[51] Razgour O, Hanmer J, Jones G (2011) Using multi-scale modelling to predict habitat suitability for species of conservation concern: The grey long-eared bat as a case study. Biol Conserv 144: 2922–2930.

[52] Phillips SJ, Anderson RP, Schapire RE (2006) Maximum entropy modelling of species geographic distributions. Ecol Modell 190: 231–259.

[53] Phillips SJ, Dudík M (2008) Modeling of species distributions with Maxent: New extensions and a comprehensive evaluation. Ecography 31: 161–175.

[54] Merow C, Smith MJ, Silander JA (2013) A practical guide to Maxent for modelling species’ distributions: What it does, and why inputs and settings matter. Ecography Accessible: 10.1111/j.1600-0587.2013.07872.x Accessed: 15 July 2013.

[55] Jiménez-Valverde A (2012) Insights into the area under the receiver operating characteristic curve (AUC) as a discrimination measure in species distribution modelling. Glob Ecol Biogeogr 21: 498–507.

[56] Warren DL, Seifert SN (2011) Ecological niche modeling in Maxent: The importance of model complexity and the performance of model selection criteria. Ecol Appli 21: 335–342. 10.1890/10-1171.1 21563566

[57] Redon M, Laque S (2010) Presence-only modelling for indicator species distribution: biodiversity monitoring in the French Alps. In: Monteil C, Paegelow W, editors. Proceedings of the 6th Spatial Analysis and Geomatics International Conference. Toulouse, France: Université de Toulouse- Le Mirail. 45–55.

[58] IBM Corp (2011) IBM SPSS Statistics for Windows. Armonk, NY: IBM.

[59] Banaszak-Cibicka W, Żmihorski M (2012) Wild bees along an urban gradient: winners and losers. J Insect Conserv 16: 331–343.

[60] Wood J, Low AB, Donaldson JS, Rebelo AG (1994) Threats to plant species diversity through urbanization and habitat fragmentation in the Cape Metropolitan Area, South Africa. Strelitzia 1: 259–74.

[61] Njoroge JB, NdaNg’ang’a PK, Natuhara Y (2013) The pattern of distribution and diversity of avifauna over an urbanizing tropical landscape. Urban Ecosys 1–15. Accessible: DOI 10.1007/s11252–013–0296–1 Accessed: 26 January 2014.

[62] Ordeñana MA, Crooks KR, Boydston EE, Fisher RN, Lyren LM, Siudyla S, Haas CD, Harris S, Hathaway SA, Turschak GM, Miles AK, Vuren DHV (2010) Effects of urbanization on carnivore species distribution and richness. J Mammal 91: 1322–1331.

[63] Duguay, S., Eigenbrod, F., Fahrig, L., 2007. Effects of surrounding urbanization on non-native flora in small forest patches. Landscape Ecol 22, 589–599.

[64] Sewell, S.R., Catterall, C.P., 1998. Bushland modification and styles of urban development: their effects on birds in south-east Queensland. Wildl Res 25, 41–63.

[65] Threlfall CG, Law B, Banks PB (2012) Sensitivity of insectivorous bats to urbanization: Implications for suburban conservation planning. Biol Cons 146: 41–52.

[66] Trollope ST, White JG, Cooke R (2009) The response of ground and bark foraging insectivorous birds across an urban–forest gradient. Landsc Urban Plan 93: 142–150.

[67] Gillies CS, St Claire CC (2008) Riparian corridors enhance movement of a forest specialist bird in a fragmented tropical forest. Proc Natl Acad Sci 105: 19774–19779. 19017794 10.1073/pnas.0803530105 PMC2604990

[68] Taylor BD, Goldingay R.L (2009) Can road-crossing structures improve population viability of an urban gliding mammal? Ecol Society 14, 13. Accessible: http://www.ecologyandsociety.org/vol14/iss2/art13/. Accessed: 16 September 2010.

[69] van Rensburg BJ, Peacock DS, Robertson MP (2009) Biotic homogenization and alien bird species along an urban gradient in South Africa. Landsc Urban Plan 92: 233–241.

[70] Abrams PA, Ginzburg LR (2000) The nature of predation: prey dependent, ratio dependent or neither? Trends Ecol Evol 15: 337–341. 10884706 10.1016/s0169-5347(00)01908-x

[71] Pimm SL (1984) The complexity and stability of ecosystems. Nature 307: 321–326.

[72] Chapin FS, Walker BH, Hobbs RJ, Hooper DU, Lawton JH (1997) Biotic Control over the Functioning of Ecosystems. Science 277, 500–504.

[73] Cooke R, Wallis R, Hogan F, White J, Webster A (2006) The diet of powerful owls (Ninox strenua) and prey availability in a continuum of habitats from disturbed urban fringe to protected forest environments in south-eastern Australia. Wildl Res 33: 199–206.

[74] Fitzsimons JA, Rose AB (2010) Diet of Powerful Owls Ninox strenua in inner City Melbourne Parks, Victoria. Australian Field Ornithology 27: 76–80.

