# Supplementary figures and images for: Simplification of Arboreal Marsupial Assemblages in Response to Increasing Urbanization
Source: PLoS One. 2014 Mar 7;9(3):e91049. doi: 10.1371/journal.pone.0091049 (PMC3946675; doi:10.1371/journal.pone.0091049)

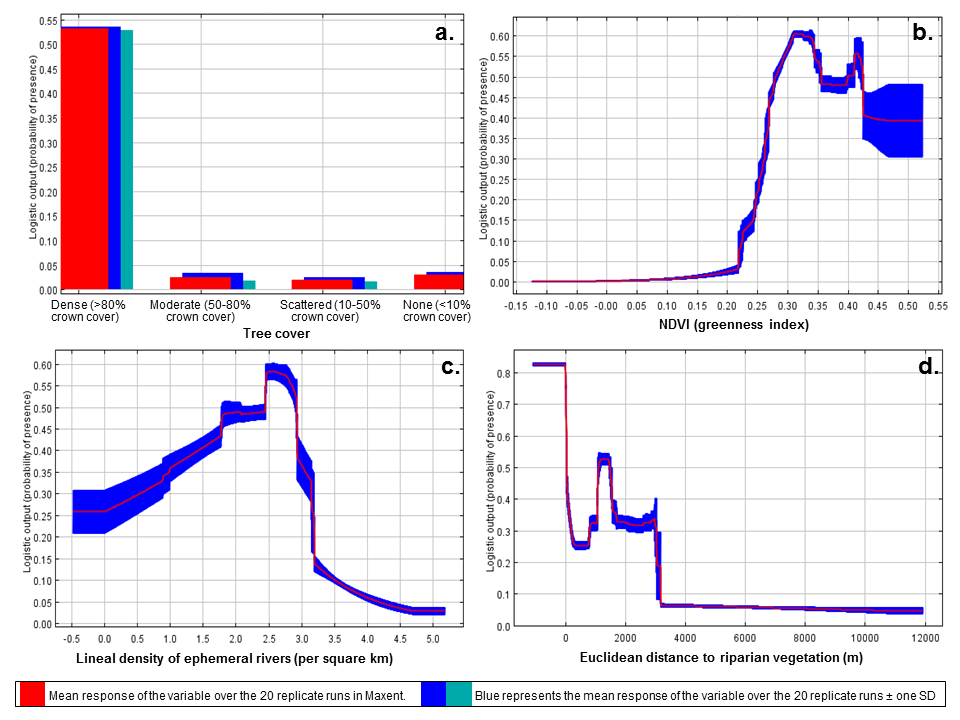

Supplement: Figure S1 — Response curves for disturbance-intolerant species. Where a. equates to the tree cover response curve; b. equates to the NDVI response curve; c. equates to the lineal density of ephemeral rivers response curve; d. equates to the Euclidean distance to riparian vegetation response curve. Red represents the mean response of the variable over the 20 replicate runs in Maxent. Blue represents the mean response of the variable over the 20 replicate runs ± one standard deviation (Categorical variables contain two shades, blue and blue/green). (TIF) [file pone.0091049.s001.tif]

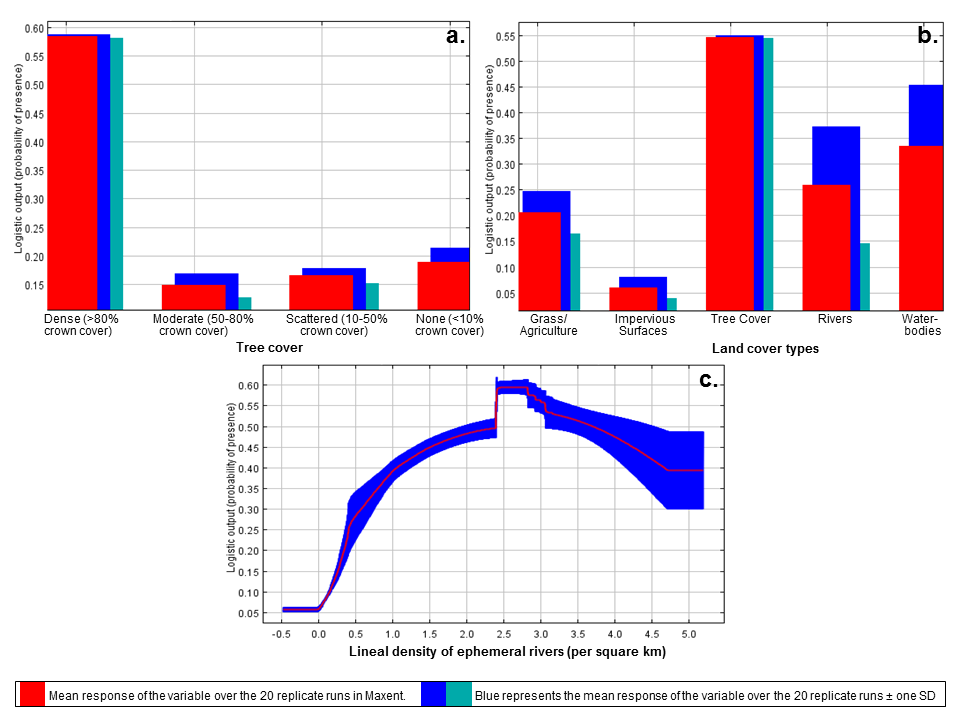

Supplement: Figure S2 — Response curves for species with moderate tolerance to disturbance. Where a. equates to the tree cover response curve; b. equates to the land cover response curve; c. equates to the lineal density of ephemeral rivers response curve. Red represents the mean response of the variable over the 20 replicate runs in Maxent. Blue represents the mean response of the variable over the 20 replicate runs ± one standard deviation (Categorical variables contain two shades, blue and blue/green). (TIF) [file pone.0091049.s002.tif]

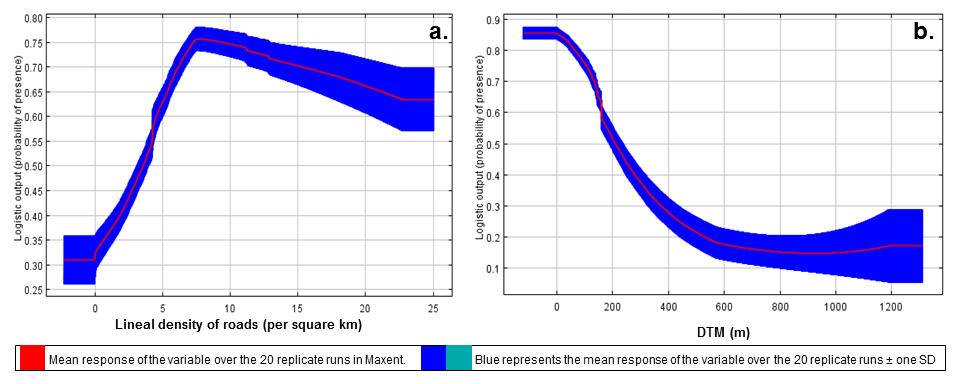

Supplement: Figure S3 — Response curves for disturbance-tolerant species. Where a. equates to the lineal density of roads response curve; b. equates to the DTM response curve; Red represents the mean response of the variable over the 20 replicate runs in Maxent. Blue represents the mean response of the variable over the 20 replicate runs ± one standard deviation. (TIF) [file pone.0091049.s003.tif]
