# Supplementary material for: Simplification of Arboreal Marsupial Assemblages in Response to Increasing Urbanization
Source: PLoS One. 2014 Mar 7;9(3):e91049. doi: 10.1371/journal.pone.0091049 (PMC3946675; doi:10.1371/journal.pone.0091049)
Supplement: Table S1 — Correlation analysis of the 11 EGV’s conducted in ENM tools. (DOCX) [file pone.0091049.s004.docx]

Table S1: Correlation analysis of the 11 EGV’s conducted in ENM tools

|  | **SPC** | **Tree Cover** | **DTM20m** | **Land Cover** | **LinDen EphRivers** | **LinDen PermRivers** | **NDVI** | **LinDen Roads** | **Riparian** | **EucDistRiparianVeg** | **LinDenRivers** |
| --- | --- | --- | --- | --- | --- | --- | --- | --- | --- | --- | --- |
| **SPC** | - | -0.03 | 0.10 | -0.01 | 0.08 | -0.02 | 0.00 | -0.08 | -0.08 | -0.04 | 0.08 |
| **Tree Cover** | - | - | -0.52 | -0.59 | -0.30 | 0.06 | -0.65 | 0.36 | -0.11 | 0.11 | -0.30 |
| **DTM20m** | - | - | - | 0.35 | 0.36 | -0.13 | 0.62 | -0.51 | -0.04 | -0.05 | 0.36 |
| **Land Cover** | - | - | - | - | 0.13 | -0.08 | 0.61 | -0.19 | 0.11 | -0.06 | 0.13 |
| **LinDen EphRivers** | - | - | - | - | - | -0.06 | 0.25 | ***-0.75****** | 0.09 | -0.43 | ***1.00****** |
| **LinDen PermRivers** | - | - | - | - | - | - | -0.08 | 0.04 | 0.08 | -0.15 | -0.06 |
| **NDVI** | - | - | - | - | - | - | - | -0.36 | 0.09 | -0.09 | 0.25 |
| **LinDen Roads** | - | - | - | - | - | - | - | - | -0.08 | 0.45 | ***-0.75****** |
| **Riparian** | - | - | - | - | - | - | - | - | - | -0.16 | 0.09 |
| **EucDistRiparianVeg** | - | - | - | - | - | - | - | - | - | - | -0.43 |
| **LinDenRivers** | - | - | - | - | - | - | - | - | - | - | - |

**Legend:** LinDen EphRivers equates to Lineal Density of Ephemeral Rivers; LinDen PermRivers equates to Lineal Density of Permanent Rivers; LinDen Roads equates to Lineal Density of Roads; EucDistRiparianVeg equates to Euclidean Distance to Riparian Vegetation; LinDenRivers equates to Lineal Density of Rivers. Bold text with asterisks highlight EGV’s with a correlation of 0.75 or above.
