# Supplementary material for: Simplification of Arboreal Marsupial Assemblages in Response to Increasing Urbanization
Source: PLoS One. 2014 Mar 7;9(3):e91049. doi: 10.1371/journal.pone.0091049 (PMC3946675; doi:10.1371/journal.pone.0091049)
Supplement: Supplementary S1 — Normalised Difference Vegetation Index (NDVI) production. (DOCX) [file pone.0091049.s005.docx]

**Supplementary 1**

***Normalised Difference Vegetation Index (NDVI) production***

Environment for Visualizing 4.7 (ENVI 4.7) was used to create a Normalised Difference Vegetation Index (NDVI). NDVI is calculated by:

$NDVI=\frac{(a_{nir}-a_{vis})}{(a_{nir} +a_{vis})}$ (1)

where $a_{vis}$ equates to the average surface reflectance in the visible (λ∼0.6) wavelengths of the spectrum and $a_{nir}$ is the average surface reflectance in the near infrared (λ∼0.8) wavelengths of the spectrum [1] - [4]. In an ecological sense, the output from the NDVI is a relative greenness index measuring the amount of live synthesising vegetation present across the surface of a landscape [5].

The NDVI for the purpose of this research was created from four high resolution SPOT 5 (Système Pour l’Observation de la Terre) images with a pixel size of 10m x10m. Two of these images were collected on 21/12/2008 and the other two on the 26/01/2009 by satellites. Before the NDVI could be created images had to have the four wavelengths specified, undergo atmospheric correction and also correct for any brightness/contrast differences between images. Corrected images were then mosaicked together, using one image as the base and a feathering distance of 100 pixels as the overlap between each image [6]. This created a near seamless image of the original four satellite images which was used to create the NDVI.

***Normalised Difference Vegetation Index (NDVI) and production of land cover layer***

The NDVI, was then used as the base layer in creating the land cover layer. One hundred and seven Regions of Interest (ROI's) were defined in each of the five habitat types of impervious surfaces, trees, grass/agriculture, water and rivers. These ROI's were used as training data during supervised classification. Supervised classification was undertaken using the maximum likelihood function, where in each pixel is given a probability that it belongs to a particular class and then assigned to the class with the highest probably [7]. The resulting output is an ascii layer that can be converted and viewed in ArcGIS 10 [8].

**References**

1. Lillesand TM, Kiefer RW, Chipman JW (2008) Remote sensing and image interpretation. United States of America: John Wiley and Sons Inc. 804 p.
2. Adams JB, Gillespie AR (2006) Remote sensing of landscapes with spectral images: a physical modelling approach. United States of America: Cambridge University Press. 378 p.
3. Cohen WB, Goward SN (2004) Landsat's role in ecological applications of remote sensing. BioScience 54: 535-545.
4. Carlson TN, Ripley DA (1997). On the relation between NDVI, fractional vegetation cover, and leaf area index. Remote Sen Environ 62: 241-252.
5. Leslie E, Sugiyama T, Ierodiaconou D, Kremer P (2010) Perceived and objectively measured greenness of neighbourhoods: Are they measuring the same thing? Landsc Urban Plan 95: 28-33.
6. Exelis Visual Information Solutions (2010) ENVI classic tutorial: mosaicking. McLean, VA: Exelis Visual Information Solutions. pp 1- 13.
7. Exelis Visual Information Solutions (2010). ENVI tutorial: classification methods. McLean, VA: Exelis Visual Information Solutions. (pp 1- 25).
8. Environmental Systems Reasearch Institute (2010) ArcGIS 10.0. Redlands, CA: Environmental Systems Research Institute.
